# 3-{[6-(4-Chloro­phen­yl)imidazo[2,1-*b*][1,3,4]thia­diazol-2-yl]meth­yl}-1,2-benzoxazole

**DOI:** 10.1107/S1600536811004582

**Published:** 2011-02-12

**Authors:** Afshan Banu, Mohamed Ziaulla, Noor Shahina Begum, Ravi S. Lamani, I. M. Khazi

**Affiliations:** a Department of Studies in Chemistry, Bangalore University, Bangalore 560 001, India; b Department of Chemistry, Karnatak University, Dharwad 580 003, India

## Abstract

In the title compound, C_18_H_11_ClN_4_OS, the benzisoxazole and imidazothia­diazole rings are inclined at an angle of 23.81 (7)° with respect to each other. The imidazothia­diazole and chloro­phenyl rings make a dihedral angle of 27.34 (3)°. In the crystal, inter­molecular C—H⋯N inter­actions generate a chain along the *c* axis and C—H⋯O inter­actions form centrosymmetric dimers resulting in an *R*_2_^2^(26) graph-set motif. Moreover, the C—H⋯N and S⋯N [3.206 (4) Å] inter­actions links the mol­ecules into *R*(7) ring motifs. The packing is further stabilized by π–π stacking inter­actions between the thia­diazole rings with a shortest centroid–centroid distance of 3.497 (3) Å. In addition, C—H⋯π inter­actions are observed in the crystal structure

## Related literature

For the preparation of the title compound see: Lamani *et al.* (2009 ▶). For the biological activity of benzisoxazole derivatives, see: Priya *et al.* (2005 ▶). For the use of five-membered heterocyclic ring 1,3,4-thia­diazo­les in the design of compounds, see: Katritzky (1984) ▶; Diamond & Sevrain (2003*a* ▶,*b* ▶); Nakao *et al.* (2002*a* ▶,*b* ▶). For related structures, see: Sun & Zhang (2009 ▶). For hydrogen-bond motifs, see: Bernstein *et al.* 1995 ▶)
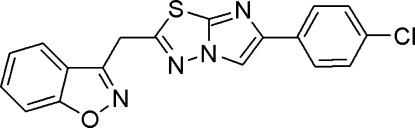


## Experimental

### 

#### Crystal data

C_18_H_11_ClN_4_OS
*M* *_r_* = 366.83
Monoclinic, 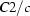

*a* = 38.419 (7) Å
*b* = 5.7761 (10) Å
*c* = 14.772 (3) Å
β = 108.004 (5)°
*V* = 3117.5 (10) Å^3^
*Z* = 8
Mo *K*α radiation
μ = 0.39 mm^−1^
*T* = 123 K
0.18 × 0.16 × 0.16 mm

#### Data collection

Bruker SMART APEX CCD detector diffractometer
Absorption correction: multi-scan Bruker Smart Apex *T*_min_ = 0.933, *T*_max_ = 0.940
8822 measured reflections
3379 independent reflections
2587 reflections with *I* > 2σ(*I*)
*R*_int_ = 0.051

#### Refinement

*R*[*F* ^2^ > 2σ(*F* ^2^)] = 0.053
*wR*(*F* ^2^) = 0.185
*S* = 1.31
3379 reflections
226 parameters
H-atom parameters constrained
Δρ_max_ = 0.74 e Å^−3^
Δρ_min_ = −0.58 e Å^−3^

### 

Data collection: *SMART* (Bruker, 1998 ▶); cell refinement: *SAINT-Plus* (Bruker, 1998 ▶); data reduction: *SAINT-Plus*; program(s) used to solve structure: *SHELXS97* (Sheldrick, 2008 ▶); program(s) used to refine structure: *SHELXL97* (Sheldrick, 2008 ▶); molecular graphics: *ORTEP-3* (Farrugia, 1997 ▶) and *CAMERON* (Watkin *et al.*, 1996) ▶; software used to prepare material for publication: *WinGX* (Farrugia, 1999 ▶).

## Supplementary Material

Crystal structure: contains datablocks global, I. DOI: 10.1107/S1600536811004582/gw2098sup1.cif

Structure factors: contains datablocks I. DOI: 10.1107/S1600536811004582/gw2098Isup2.hkl

Additional supplementary materials:  crystallographic information; 3D view; checkCIF report

## Figures and Tables

**Table 1:** Hydrogen-bond geometry (Å, °) *Cg*1 and *Cg*2 are the centroids of the C7–C12 and C13–C18 rings, respectively.

*D*—H⋯*A*	*D*—H	H⋯*A*	*D*⋯*A*	*D*—H⋯*A*
C9—H9⋯O1^i^	0.93	2.39	3.232 (4)	150
C2—H2*B*⋯N1^ii^	0.97	2.49	3.352 (4)	148
C5—H5⋯N1^iii^	0.93	2.60	3.478 (4)	157
C17—H17⋯*Cg*1^iv^	0.93	2.78	3.470 (4)	131
C11—H11⋯*Cg*2^iv^	0.93	2.93	3.548 (4)	126

Symmetry codes: (i) 
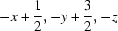
; (ii) 
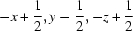
; (iii) 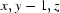; (iv) 
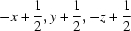
.

**Fig. 1.**
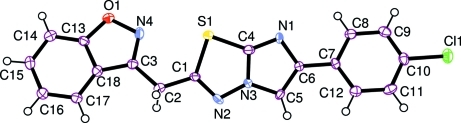
ORTEP (Farrugia, 1997) view of the title compound, showing 50% probability ellipsoids and the atom numbering scheme.

**Fig. 2.**
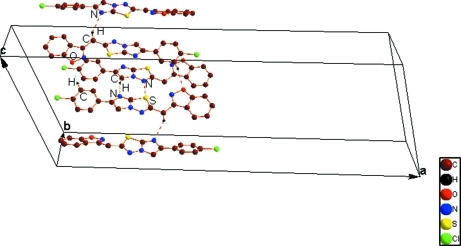
A unit cell packing of the title compound depicting the C—H···N, C—H···O and S···N intermolecular interactions with dotted lines. H-atoms not involved in hydrogen bonding have been excluded.

## supplementary crystallographic information

### Comment

Imidazo[2,1-*b*][1,3,4]thiadiazole derivatives are reported to possess diverse pharmacological properties such as anticancer, antitubercular, antibacterial, antifungal, anticonvulsant, analgesic and antisecretory activities. Moreover, the  are known to possess important biological activities (Priya *et al.*, 2005) and are useful in different therapies. Amongst them, five membered heterocyclic ring 1,3,4-thiadiazoles find wide application in designing compounds possessing useful properties (Katritzky *et al.*, 1984; Diamond & Sevrain, 2003*a*,*b*; Nakao *et al.*, 2002*a*,*b*). Due to the increasing importance of these heterocycles in biological and pharmaceutical fields, new chemical entities were synthesized by incorporating active pharmacophores in a single molecular frame work so as to enhance their biological activities.In the title compound, the benzisoxazole (O1/N4/C3/C13–18) and imidazothiadiazole (S1/N1—N3/C1/C4—C6) rings are individually planar similar to those reported earlier (Sun & Zhang, 2009) with maximum deviations of 0.038 (3)Å for C1 and 0.016 (3)Å for C3 respectively. The mean planes of the benzisoxazole and imidazothiadiazole are inclined at an angle 23.81 (7)° with each other. The imidazothiadiazole and chlorophenyl rings make a dihedral angle of 27.34 (3)°. The thiadiazole moiety displays differences in the bond lengths between S1—C1/S1—C4 [1.756 (3)/1.736 (3)]. This can be attributed to the resonance effects of the imidazole ring which is stronger than that due to thiadiazole group. The crystal structure is stabilized by intermolecular C—H···N, C—H···O and S···N interactions. The C—H···N interaction generates chain like pattern along c axis. The C—H···O interaction forms centrosymmetric head-to-head dimers about inversion centers corresponding to R22(26) graph set motif (Bernstein *et al.*, 1995). The C—H···N interaction along with S···N [3.206 (4) Å]interaction results in a ring motif with a graph set *R*(7). The molecular packing is further stabilized by π-π stacking interactions between thiadiazole rings (*Cg*3: centroid of S1/C1/N2/N3/C4) with the shortest centroid—centroid distance 3.497 (3) Å. In addition, π-ring interactions of the type C—H···Cg (*Cg* being the centroids of rings C7—C12 and C13—C18) are also observed in the crystal structure; details have been given in Table 1.

### Experimental

The title compound was synthesized by following the procedure reported earlier (Lamani *et al.*, 2009).

### Refinement

The H atoms were placed at calculated positions in the riding model approximation with aromatic C—H = 0.93Å and methylene C—H = 0.97 Å, and *U*_iso_(H) = 1.2*U*_eq_(N/C).

### Crystal data

**Table:**

C_18_H_11_ClN_4_OS	*F*(000) = 1504
*M**_r_* = 366.83	*D*_x_ = 1.563 Mg m^−^^3^
Monoclinic, *C*2/*c*	Mo *K*α radiation, λ = 0.71073 Å
Hall symbol: -C 2yc	Cell parameters from 3379 reflections
*a* = 38.419 (7) Å	θ = 2.2–27.0°
*b* = 5.7761 (10) Å	µ = 0.39 mm^−^^1^
*c* = 14.772 (3) Å	*T* = 123 K
β = 108.004 (5)°	Block, yellow
*V* = 3117.5 (10) Å^3^	0.18 × 0.16 × 0.16 mm
*Z* = 8	

### Data collection

**Table:**

Bruker SMART APEX CCD detector diffractometer	3379 independent reflections
Radiation source: fine-focus sealed tube	2587 reflections with *I* > 2σ(*I*)
graphite	*R*_int_ = 0.051
ω scans	θ_max_ = 27.0°, θ_min_ = 2.2°
Absorption correction: multi-scan Bruker Smart Apex	*h* = −46→48
*T*_min_ = 0.933, *T*_max_ = 0.940	*k* = −6→7
8822 measured reflections	*l* = −18→14

### Refinement

**Table:**

Refinement on *F*^2^	Primary atom site location: structure-invariant direct methods
Least-squares matrix: full	Secondary atom site location: difference Fourier map
*R*[*F*^2^ > 2σ(*F*^2^)] = 0.053	Hydrogen site location: inferred from neighbouring sites
*wR*(*F*^2^) = 0.185	H-atom parameters constrained
*S* = 1.31	*w* = 1/[σ^2^(*F*_o_^2^) + (0.0894*P*)^2^] where *P* = (*F*_o_^2^ + 2*F*_c_^2^)/3
3379 reflections	(Δ/σ)_max_ < 0.001
226 parameters	Δρ_max_ = 0.74 e Å^−^^3^
0 restraints	Δρ_min_ = −0.58 e Å^−^^3^

### Special details

**Table:**

Experimental. The compound was synthesized by following the procedure given in Lamani *et al.*, (2009)
Geometry. All s.u.'s (except the s.u. in the dihedral angle between two l.s. planes) are estimated using the full covariance matrix. The cell s.u.'s are taken into account individually in the estimation of s.u.'s in distances, angles and torsion angles; correlations between s.u.'s in cell parameters are only used when they are defined by crystal symmetry. An approximate (isotropic) treatment of cell s.u.'s is used for estimating s.u.'s involving l.s. planes.
Refinement. Refinement of *F*^2^ against ALL reflections. The weighted *R*-factor *wR* and goodness of fit *S* are based on *F*^2^, conventional *R*-factors *R* are based on *F*, with *F* set to zero for negative *F*^2^. The threshold expression of *F*^2^ > 2σ(*F*^2^) is used only for calculating *R*-factors(gt) *etc*. and is not relevant to the choice of reflections for refinement. *R*-factors based on *F*^2^ are statistically about twice as large as those based on *F*, and *R*- factors based on ALL data will be even larger.

### Fractional atomic coordinates and isotropic or equivalent isotropic displacement parameters (Å^2^)

**Table:**

	*x*	*y*	*z*	*U*_iso_*/*U*_eq_	
C1	0.19871 (8)	0.2236 (5)	0.1424 (2)	0.0151 (6)	
C2	0.16451 (8)	0.1564 (5)	0.1657 (2)	0.0183 (7)	
H2A	0.1565	0.0071	0.1366	0.022*	
H2B	0.1711	0.1355	0.2341	0.022*	
C3	0.13263 (8)	0.3171 (5)	0.1363 (2)	0.0166 (7)	
C4	0.25323 (8)	0.4023 (5)	0.1246 (2)	0.0160 (7)	
C5	0.28417 (8)	0.0835 (6)	0.1177 (2)	0.0167 (6)	
H5	0.2910	−0.0704	0.1157	0.020*	
C6	0.30436 (8)	0.2790 (5)	0.1142 (2)	0.0149 (6)	
C7	0.34193 (8)	0.2930 (5)	0.1098 (2)	0.0148 (7)	
C8	0.35464 (9)	0.4896 (5)	0.0746 (2)	0.0181 (7)	
H8	0.3389	0.6139	0.0527	0.022*	
C9	0.39034 (9)	0.5024 (5)	0.0718 (2)	0.0192 (7)	
H9	0.3986	0.6340	0.0484	0.023*	
C10	0.41322 (8)	0.3176 (6)	0.1039 (2)	0.0183 (7)	
C11	0.40183 (9)	0.1192 (6)	0.1396 (2)	0.0206 (7)	
H11	0.4178	−0.0038	0.1616	0.025*	
C12	0.36610 (8)	0.1082 (6)	0.1418 (2)	0.0178 (7)	
H12	0.3580	−0.0244	0.1649	0.021*	
C13	0.07777 (9)	0.4766 (5)	0.1117 (2)	0.0176 (7)	
C14	0.04157 (9)	0.5159 (6)	0.1076 (2)	0.0209 (7)	
H14	0.0292	0.6519	0.0834	0.025*	
C15	0.02526 (9)	0.3370 (6)	0.1424 (2)	0.0219 (7)	
H15	0.0010	0.3519	0.1407	0.026*	
C16	0.04440 (8)	0.1336 (6)	0.1800 (3)	0.0212 (7)	
H16	0.0326	0.0188	0.2036	0.025*	
C17	0.08036 (8)	0.1001 (6)	0.1828 (2)	0.0178 (7)	
H17	0.0928	−0.0352	0.2074	0.021*	
C18	0.09718 (8)	0.2764 (5)	0.1474 (2)	0.0166 (7)	
O1	0.09977 (6)	0.6281 (4)	0.08271 (17)	0.0222 (5)	
N1	0.28445 (7)	0.4813 (4)	0.11762 (19)	0.0161 (6)	
N2	0.22129 (7)	0.0628 (5)	0.13533 (19)	0.0171 (6)	
N3	0.25177 (7)	0.1673 (4)	0.12454 (19)	0.0158 (6)	
N4	0.13496 (7)	0.5207 (5)	0.1006 (2)	0.0208 (6)	
S1	0.21240 (2)	0.51078 (13)	0.13431 (6)	0.0182 (2)	
Cl1	0.45843 (2)	0.33439 (15)	0.10073 (6)	0.0267 (3)	

### Atomic displacement parameters (Å^2^)

**Table:**

	*U*^11^	*U*^22^	*U*^33^	*U*^12^	*U*^13^	*U*^23^
C1	0.0141 (14)	0.0126 (15)	0.0175 (16)	0.0005 (12)	0.0032 (13)	0.0015 (12)
C2	0.0170 (15)	0.0154 (16)	0.0248 (18)	0.0016 (12)	0.0095 (14)	0.0034 (13)
C3	0.0197 (15)	0.0141 (15)	0.0155 (16)	−0.0028 (12)	0.0049 (13)	−0.0002 (12)
C4	0.0202 (15)	0.0121 (15)	0.0152 (16)	0.0008 (12)	0.0047 (13)	−0.0006 (12)
C5	0.0152 (14)	0.0138 (15)	0.0214 (16)	0.0032 (12)	0.0062 (13)	0.0002 (13)
C6	0.0131 (14)	0.0162 (16)	0.0153 (16)	−0.0011 (12)	0.0042 (13)	0.0011 (12)
C7	0.0137 (14)	0.0156 (16)	0.0159 (16)	−0.0034 (12)	0.0054 (12)	−0.0023 (12)
C8	0.0206 (16)	0.0157 (16)	0.0191 (17)	0.0021 (12)	0.0078 (14)	0.0006 (12)
C9	0.0252 (17)	0.0161 (17)	0.0176 (17)	−0.0052 (13)	0.0086 (14)	−0.0021 (12)
C10	0.0142 (14)	0.0231 (17)	0.0179 (17)	−0.0048 (12)	0.0051 (13)	−0.0056 (13)
C11	0.0231 (16)	0.0177 (17)	0.0220 (18)	0.0049 (13)	0.0084 (14)	−0.0019 (13)
C12	0.0193 (15)	0.0169 (16)	0.0178 (17)	0.0012 (13)	0.0067 (13)	0.0014 (13)
C13	0.0190 (16)	0.0158 (16)	0.0204 (17)	−0.0004 (12)	0.0096 (14)	0.0009 (13)
C14	0.0194 (16)	0.0200 (17)	0.0220 (18)	0.0035 (13)	0.0042 (14)	0.0000 (13)
C15	0.0177 (16)	0.0283 (19)	0.0209 (18)	−0.0017 (13)	0.0077 (14)	−0.0013 (14)
C16	0.0149 (15)	0.0216 (17)	0.0276 (19)	−0.0029 (13)	0.0075 (14)	0.0010 (14)
C17	0.0172 (15)	0.0163 (16)	0.0202 (17)	0.0005 (12)	0.0060 (13)	0.0023 (13)
C18	0.0136 (14)	0.0178 (16)	0.0183 (16)	0.0011 (12)	0.0049 (13)	−0.0003 (13)
O1	0.0200 (11)	0.0167 (12)	0.0325 (14)	0.0044 (9)	0.0120 (11)	0.0055 (10)
N1	0.0114 (12)	0.0167 (14)	0.0206 (14)	0.0006 (10)	0.0054 (11)	−0.0008 (11)
N2	0.0127 (12)	0.0178 (14)	0.0211 (15)	−0.0055 (10)	0.0059 (11)	0.0001 (11)
N3	0.0174 (13)	0.0115 (13)	0.0189 (15)	−0.0012 (10)	0.0059 (11)	0.0013 (10)
N4	0.0147 (13)	0.0244 (16)	0.0251 (16)	0.0015 (11)	0.0087 (12)	0.0010 (12)
S1	0.0184 (4)	0.0124 (4)	0.0257 (5)	−0.0008 (3)	0.0096 (4)	−0.0007 (3)
Cl1	0.0177 (4)	0.0329 (5)	0.0318 (5)	−0.0021 (3)	0.0110 (4)	−0.0057 (4)

### Geometric parameters (Å, °)

**Table:**

C1—N2	1.297 (4)	C9—C10	1.371 (4)
C1—C2	1.508 (4)	C9—H9	0.9300
C1—S1	1.756 (3)	C10—C11	1.387 (5)
C2—C3	1.490 (4)	C10—Cl1	1.755 (3)
C2—H2A	0.9700	C11—C12	1.384 (4)
C2—H2B	0.9700	C11—H11	0.9300
C3—N4	1.304 (4)	C12—H12	0.9300
C3—C18	1.440 (4)	C13—O1	1.374 (4)
C4—N1	1.316 (4)	C13—C14	1.392 (4)
C4—N3	1.358 (4)	C13—C18	1.389 (4)
C4—S1	1.736 (3)	C14—C15	1.387 (5)
C5—C6	1.380 (4)	C14—H14	0.9300
C5—N3	1.369 (4)	C15—C16	1.406 (5)
C5—H5	0.9300	C15—H15	0.9300
C6—N1	1.406 (4)	C16—C17	1.383 (4)
C6—C7	1.468 (4)	C16—H16	0.9300
C7—C12	1.398 (4)	C17—C18	1.392 (4)
C7—C8	1.398 (4)	C17—H17	0.9300
C8—C9	1.387 (4)	O1—N4	1.436 (3)
C8—H8	0.9300	N2—N3	1.370 (3)
			
N2—C1—C2	119.1 (3)	C12—C11—C10	118.5 (3)
N2—C1—S1	116.7 (2)	C12—C11—H11	120.7
C2—C1—S1	124.0 (2)	C10—C11—H11	120.7
C3—C2—C1	117.9 (3)	C11—C12—C7	121.1 (3)
C3—C2—H2A	107.8	C11—C12—H12	119.4
C1—C2—H2A	107.8	C7—C12—H12	119.4
C3—C2—H2B	107.8	O1—C13—C14	125.8 (3)
C1—C2—H2B	107.8	O1—C13—C18	109.8 (3)
H2A—C2—H2B	107.2	C14—C13—C18	124.4 (3)
N4—C3—C18	112.3 (3)	C13—C14—C15	115.0 (3)
N4—C3—C2	121.8 (3)	C13—C14—H14	122.5
C18—C3—C2	125.9 (3)	C15—C14—H14	122.5
N1—C4—N3	112.7 (3)	C14—C15—C16	121.9 (3)
N1—C4—S1	138.5 (3)	C14—C15—H15	119.1
N3—C4—S1	108.8 (2)	C16—C15—H15	119.1
C6—C5—N3	104.3 (3)	C17—C16—C15	121.6 (3)
C6—C5—H5	127.8	C17—C16—H16	119.2
N3—C5—H5	127.8	C15—C16—H16	119.2
C5—C6—N1	111.2 (3)	C16—C17—C18	117.5 (3)
C5—C6—C7	128.2 (3)	C16—C17—H17	121.3
N1—C6—C7	120.6 (3)	C18—C17—H17	121.3
C12—C7—C8	118.3 (3)	C17—C18—C13	119.7 (3)
C12—C7—C6	120.2 (3)	C17—C18—C3	136.7 (3)
C8—C7—C6	121.5 (3)	C13—C18—C3	103.7 (3)
C9—C8—C7	121.1 (3)	C13—O1—N4	107.6 (2)
C9—C8—H8	119.4	C4—N1—C6	103.5 (2)
C7—C8—H8	119.4	C1—N2—N3	108.1 (3)
C10—C9—C8	118.8 (3)	C4—N3—N2	118.5 (3)
C10—C9—H9	120.6	C4—N3—C5	108.4 (3)
C8—C9—H9	120.6	N2—N3—C5	133.0 (3)
C9—C10—C11	122.1 (3)	C3—N4—O1	106.6 (2)
C9—C10—Cl1	118.8 (2)	C4—S1—C1	87.82 (14)
C11—C10—Cl1	119.1 (2)		
			
N2—C1—C2—C3	−155.9 (3)	O1—C13—C18—C3	−0.9 (3)
S1—C1—C2—C3	30.1 (4)	C14—C13—C18—C3	179.8 (3)
C1—C2—C3—N4	−7.1 (5)	N4—C3—C18—C17	−177.9 (4)
C1—C2—C3—C18	176.2 (3)	C2—C3—C18—C17	−0.9 (6)
N3—C5—C6—N1	−0.8 (3)	N4—C3—C18—C13	1.6 (4)
N3—C5—C6—C7	177.9 (3)	C2—C3—C18—C13	178.6 (3)
C5—C6—C7—C12	−22.8 (5)	C14—C13—O1—N4	179.2 (3)
N1—C6—C7—C12	155.8 (3)	C18—C13—O1—N4	0.0 (3)
C5—C6—C7—C8	157.7 (3)	N3—C4—N1—C6	−0.8 (3)
N1—C6—C7—C8	−23.7 (4)	S1—C4—N1—C6	178.9 (3)
C12—C7—C8—C9	−0.3 (5)	C5—C6—N1—C4	1.0 (3)
C6—C7—C8—C9	179.2 (3)	C7—C6—N1—C4	−177.8 (3)
C7—C8—C9—C10	0.2 (5)	C2—C1—N2—N3	−173.3 (3)
C8—C9—C10—C11	−0.3 (5)	S1—C1—N2—N3	1.1 (3)
C8—C9—C10—Cl1	−179.8 (2)	N1—C4—N3—N2	177.4 (3)
C9—C10—C11—C12	0.5 (5)	S1—C4—N3—N2	−2.5 (3)
Cl1—C10—C11—C12	180.0 (2)	N1—C4—N3—C5	0.3 (4)
C10—C11—C12—C7	−0.7 (5)	S1—C4—N3—C5	−179.5 (2)
C8—C7—C12—C11	0.6 (5)	C1—N2—N3—C4	0.9 (4)
C6—C7—C12—C11	−179.0 (3)	C1—N2—N3—C5	177.1 (3)
O1—C13—C14—C15	−179.3 (3)	C6—C5—N3—C4	0.3 (3)
C18—C13—C14—C15	−0.1 (5)	C6—C5—N3—N2	−176.1 (3)
C13—C14—C15—C16	0.9 (5)	C18—C3—N4—O1	−1.7 (4)
C14—C15—C16—C17	−1.1 (5)	C2—C3—N4—O1	−178.8 (3)
C15—C16—C17—C18	0.4 (5)	C13—O1—N4—C3	1.1 (3)
C16—C17—C18—C13	0.4 (5)	N1—C4—S1—C1	−177.4 (4)
C16—C17—C18—C3	179.8 (4)	N3—C4—S1—C1	2.4 (2)
O1—C13—C18—C17	178.7 (3)	N2—C1—S1—C4	−2.1 (3)
C14—C13—C18—C17	−0.6 (5)	C2—C1—S1—C4	172.1 (3)

### Hydrogen-bond geometry (Å, °)

**Table:**

Cg1 and Cg2 are the centroids of the C7–C12 and C13–C18 rings, respectively.

**Table:**

*D*—H···*A*	*D*—H	H···*A*	*D*···*A*	*D*—H···*A*
C9—H9···O1^i^	0.93	2.39	3.232 (4)	150
C2—H2B···N1^ii^	0.97	2.49	3.352 (4)	148
C5—H5···N1^iii^	0.93	2.60	3.478 (4)	157
C17—H17···Cg1^iv^	0.93	2.78	3.470 (4)	131
C11—H11···Cg2^iv^	0.93	2.93	3.548 (4)	126

Symmetry codes: (i) −*x*+1/2, −*y*+3/2, −*z*; (ii) −*x*+1/2, *y*−1/2, −*z*+1/2; (iii) *x*, *y*−1, *z*; (iv) −*x*+1/2, *y*+1/2, −*z*+1/2.
